# Magnitude of Survival Benefit Associated With Tyrosine Kinase Inhibitors in Advanced Thyroid Carcinoma: A Systematic Review and Meta-Analysis Incorporating Updated Long-Term Evidence From SELECT (Lenvatinib) and COSMIC-311 (Cabozantinib) Trials

**DOI:** 10.14740/wjon2842

**Published:** 2026-09-04

**Authors:** Pushpita Roy, Dipaloke Banik, Forhad Chowdhury, Md Abu Bakar Siddique

**Affiliations:** a College of Medicine and Veterinary Medicine, Edinburgh Medical School, The University of Edinburgh, Edinburgh, UK; b Department of Public Health, SUNY Downstate Health Sciences University, Brooklyn, NY, USA; c Nuffield Department of Medicine, University of Oxford, Oxford, UK; d Department of Biological Sciences, St. John’s University, Queens, NY, USA

**Keywords:** Oncology, Thyroid, Therapeutics, Targeted therapy, Survival outcome, Efficacy, Systematic review

## Abstract

**Background:**

Tyrosine kinase inhibitors (TKIs) have transformed the management of advanced thyroid carcinoma. However, newer agents and updated long-term evidence from pivotal trials have not been comprehensively synthesized. This systematic review and meta-analysis integrates recent randomized evidence, including donafenib and nintedanib, with qualitative synthesis of updated SELECT (lenvatinib) and COSMIC-311 (cabozantinib) analyses, alongside separate quantitative evaluations of placebo-controlled and dose-comparison trials.

**Methods:**

PubMed/MEDLINE, Embase, Cochrane CENTRAL, Google Scholar, and reference lists were systematically searched for randomized controlled trials evaluating TKIs in advanced thyroid carcinoma. Eighteen studies were included in the systematic review, of which 14 publications contributed to the meta-analysis. Updated SELECT and COSMIC-311 analyses were synthesized qualitatively to avoid duplication of patient populations. For trials reporting progression-free survival (PFS) and overall survival (OS) in separate publications, the most appropriate publication was selected for each outcome, resulting in risk-of-bias assessment of 13 unique randomized trials using the Cochrane RoB 2 tool. Random-effects meta-analyses were performed using SAS version 9.4.

**Results:**

Thirteen randomized controlled trials involving 2,671 patients were quantitatively analyzed. Compared with placebo, TKIs significantly improved PFS (hazard ratio (HR) = 0.36; 95% confidence interval (CI), 0.26–0.50; P < 0.001), reducing the risk of disease progression or death by approximately 64%, although heterogeneity was substantial (I^2^ = 83.8%). Separate dose-comparison analyses of three trials demonstrated no significant advantage of higher-dose regimens (HR = 1.18; 95% CI, 0.87–1.60). TKIs also significantly improved OS (HR = 0.78; 95% CI, 0.67–0.91; P = 0.002), corresponding to a 22% reduction in mortality risk, with no heterogeneity (I^2^ = 0%). Lenvatinib demonstrated the greatest PFS benefit, followed by cabozantinib. Updated SELECT and COSMIC-311 analyses confirmed durable efficacy across extended follow-up and multiple patient subgroups. No significant publication bias was detected.

**Conclusions:**

TKIs significantly improve PFS and OS in advanced thyroid carcinoma, supporting their role in contemporary clinical management. Lenvatinib demonstrated the greatest efficacy, while updated long-term evidence reinforced the durable benefits of lenvatinib and cabozantinib. Although placebo-controlled trials consistently favored TKIs, dose-comparison trials did not demonstrate superiority of higher-dose regimens. Further randomized studies with longer follow-up and biomarker-guided treatment strategies are warranted.

## Introduction

The World Health Organization (WHO) International Agency for Research on Cancer, using the GOBOCAN database, reports that thyroid cancer is the ninth most common cancer globally and the most prevalent endocrine malignancy, representing 3% of all cancer diagnoses [1–3]. The more advanced strategies for diagnosing thyroid conditions have contributed to the rising incidence of early-stage well-differentiated cancers; however, there has also been an increase in advanced metastatic cases over the past few decades [4].

Thyroid cancer encompasses a significant spectrum of malignancies, forming the largest section in the fifth edition of the WHO Classification of Endocrine and Neuroendocrine Tumors [5]. The primary cell types from which thyroid malignancies originate are parafollicular C and follicular thyroid cells [6–8]. Medullary thyroid cancers are derived from parafollicular C cells, which comprise a tiny portion of the overall incidence of thyroid malignancies (2–3%) [9]. Nevertheless, unlike other types of thyroid carcinomas, which tend to have a relatively indolent progression, medullary thyroid carcinoma (MTC) accounts for a disproportionately large number of fatalities [10]. Most thyroid malignancies originate from follicular cells and are subdivided into follicular thyroid cancer (FTC, 6–10%), papillary thyroid cancer (PTC, 65–93%), poorly differentiated thyroid cancer (PDTC, 0.3–6.7%), Hurthle cell cancer (currently known as oncocytic carcinoma of thyroid) [11], and anaplastic thyroid cancer [12]. FTC and PTC are differentiated thyroid carcinomas (DTCs), and anaplastic carcinoma is the most aggressive form of malignancy among the follicular-derived cancers [9].

Selecting treatment strategies for thyroid malignancy largely depends on individual patient’s histopathological characteristics and preoperative risk assessment [6]. Surgery is typically the first-line approach for most patients, with the extent of surgical intervention (such as lobectomy, total thyroidectomy with or without central neck dissection, and extended resection) is determined by both the radiological and histological status of the tumor [13]. Considering the impact of surgery on quality of life and financial considerations, non-surgical options are gaining increased interest [14, 15]. Active surveillance, a risk- and cost-effective strategy, is gaining recognition and is a reassuring sign of progress in treatment strategies mentioned by Surveillance, Epidemiology and End Results-Medicare database-based study [16]. Radioactive iodine (RAI) therapy is commonly utilized to prevent or treat recurrent disease; however, there has been a noticeable increase in radioactive iodine-refractory (RAI-R) cases [17]. Among these, patients with targetable, locally oligometastatic disease may be treated with surgery combined with external beam radiation therapy (EBRT) [13]. For patients with locally advanced and/or symptomatic metastatic RAI-R disease, targeted therapies are chosen as the preferred treatment option demonstrating an increasing level of efficacy and promising outcomes [13, 17–25].

Papillary and follicular thyroid carcinomas generally respond favorably to conventional treatment modalities, including surgery, RAI therapy, and thyroid-stimulating hormone (TSH) suppression [26]. However, some metastatic lesions may undergo dedifferentiation during the disease course and lose their ability to concentrate iodine, resulting in radioiodine-refractory differentiated thyroid cancer (RR-DTC), which is associated with a significantly poorer prognosis [15]. In contrast, MTC and anaplastic thyroid carcinoma (ATC) are inherently resistant to RAI therapy. Consequently, radioiodine-refractory thyroid cancer has emerged as an increasingly important therapeutic challenge and a growing area of research interest within endocrine oncology.

A potential challenge in the treatment of thyroid malignancy is the increasing incidence of RAI-R thyroid cancer. Until years ago, conventional forms of treatment like external beam therapy and chemotherapy showed evidence of more severe toxicities, adverse events, low efficacy, identifying these as palliative forms of management [27]. Additionally, it showed a transient effect with no survival prolongation using either single form of treatment or both in combination [28, 29]. Managing RAI-R thyroid malignancy has increasingly given rise to the development of newer varieties of drugs in the recent past. Tyrosine-kinase receptors have been identified as one of the potential targets in thyroid malignancy, which are responsible for tumor growth and angiogenesis [30]. The drugs recently evaluated for targeting these receptors have demonstrated strong evidence of blocking tyrosine kinase receptors, along with other kinases, ultimately impeding cell proliferation and tumor transformation [30]; and many of them have been approved by Food and Drug Administration [8].

The novelty of this systematic review and meta-analysis includes its incorporation of recently published evidence that has not been comprehensively synthesized in previous reviews [31] of targeted therapies for thyroid carcinoma. In addition to evaluating established tyrosine kinase inhibitors (TKIs), this review includes newer therapeutic agents such as donafenib [32] and nintedanib [33], thereby expanding the current evidence base. Furthermore, updated follow-up analyses from the pivotal SELECT (lenvatinib) and COSMIC-311 (cabozantinib) trials were reviewed and presented qualitatively through descriptive data extraction to provide a more contemporary understanding of the durability and consistency of treatment benefit, while avoiding duplication of patient populations in quantitative meta-analysis.

While both progression-free survival (PFS) and overall survival (OS) were evaluated, this review places particular emphasis on PFS, as it represents the primary efficacy endpoint in most pivotal targeted therapy trials and serves as the principal basis for regulatory approval and clinical decision-making in advanced thyroid carcinoma. Moreover, efficacy outcomes were assessed through separate analyses of targeted therapy versus placebo and targeted therapy with different dosage comparisons. The dose-versus-dose analyses were performed independently to explore whether higher-dose regimens confer superior efficacy over lower-dose strategies, thereby providing additional evidence to inform treatment optimization.

By integrating newly available agents, updated long-term evidence, dedicated analyses of PFS and OS outcomes, qualitative synthesis of updated follow-up analyses, and separate evaluations of placebo-controlled and dose-comparison trials, this review may complement the existing literature and support future research and clinical decision-making in this evolving field.

## Materials and Methods

### Eligibility criteria

Eligibility criteria were established *a priori* using the Population, Intervention, Comparator, Outcome, and Study Design (PICOS) framework. Studies involving adult patients diagnosed with any form of thyroid carcinoma, including papillary thyroid carcinoma, follicular thyroid carcinoma, MTC, ATC, and other recognized thyroid cancer subtypes, were considered eligible for inclusion. Studies involving pediatric populations or malignancies other than primary thyroid carcinoma were excluded.

The intervention of interest was TKIs administered for the treatment of thyroid carcinoma. Eligible studies included those evaluating targeted agents either against placebo or against another targeted therapeutic regimen. Consequently, both targeted therapy versus placebo trials and targeted therapy dosage comparison trials were included in the review. Studies investigating only conventional treatment modalities, such as surgery, RAI therapy, chemotherapy, or radiotherapy without a targeted therapy arm, were excluded. Similarly, studies lacking a targeted therapy intervention in the experimental arm were not considered eligible.

The outcomes of interest were PFS and OS. Studies reporting either or both efficacy outcomes were considered eligible for inclusion in the review and were synthesized according to the available outcome data. Studies that did not report at least one of these predefined efficacy or safety outcomes were excluded from the review.

To ensure a high level of evidence, only randomized controlled trials and prospective clinical trials were included. Non-randomized controlled studies, observational studies, case reports, case series, conference abstracts without full-text publication, review articles, editorials, and commentaries were excluded. Additionally, only studies published in English and available as full-text articles were considered eligible for inclusion.

### Search strategy

A comprehensive search strategy was developed based on the predefined Population, Intervention, Comparator, and Outcome (PICO) framework to ensure a systematic and reproducible literature search. The eligibility criteria were established prior to study selection and implemented within the Covidence platform in accordance with the Preferred Reporting Items for Systematic Reviews and Meta-Analyses (PRISMA) guidelines. A comprehensive literature search was conducted using PubMed/MEDLINE, Embase, the Cochrane Central Register of Controlled Trials (CENTRAL), and Google Scholar, with manual screening of the reference lists of eligible studies to identify additional relevant publications. The literature search was conducted from database inception through September 2026.

Following database retrieval, duplicate records were identified and removed. Subsequently, title and abstract screening were conducted in Covidence, followed by full-text assessment of potentially eligible studies using the predefined inclusion and exclusion criteria.

The search strategy was designed to identify studies evaluating the efficacy of targeted therapies in thyroid carcinoma. Search terms related to the population included thyroid neoplasm, thyroid cancer, thyroid carcinoma, thyroid malignancy, differentiated thyroid cancer, papillary thyroid cancer, follicular thyroid cancer, medullary thyroid cancer, anaplastic thyroid cancer, and poorly differentiated thyroid cancer. Intervention-related search terms included both generic intervention terms and specific therapeutic agents to maximize search sensitivity. Generic terms included “targeted therapy,” “molecular targeted therapy,” “tyrosine kinase inhibitor,” “multikinase inhibitor,” and “protein kinase inhibitor,” together with individual TKIs names relevant to thyroid cancer including sorafenib, lenvatinib, vandetanib, cabozantinib, donafenib, nintedanib, anlotinib, apatinib, axitinib, selpercatinib, pralsetinib, dabrafenib, and trametinib. Comparator-related terms included placebo, standard therapy, surgery, radioactive iodine, chemotherapy, and radiotherapy. Outcome-related terms included PFS, OS, objective response rate, disease control rate, adverse effects, toxicity, and quality of life.

**Figure 1:**
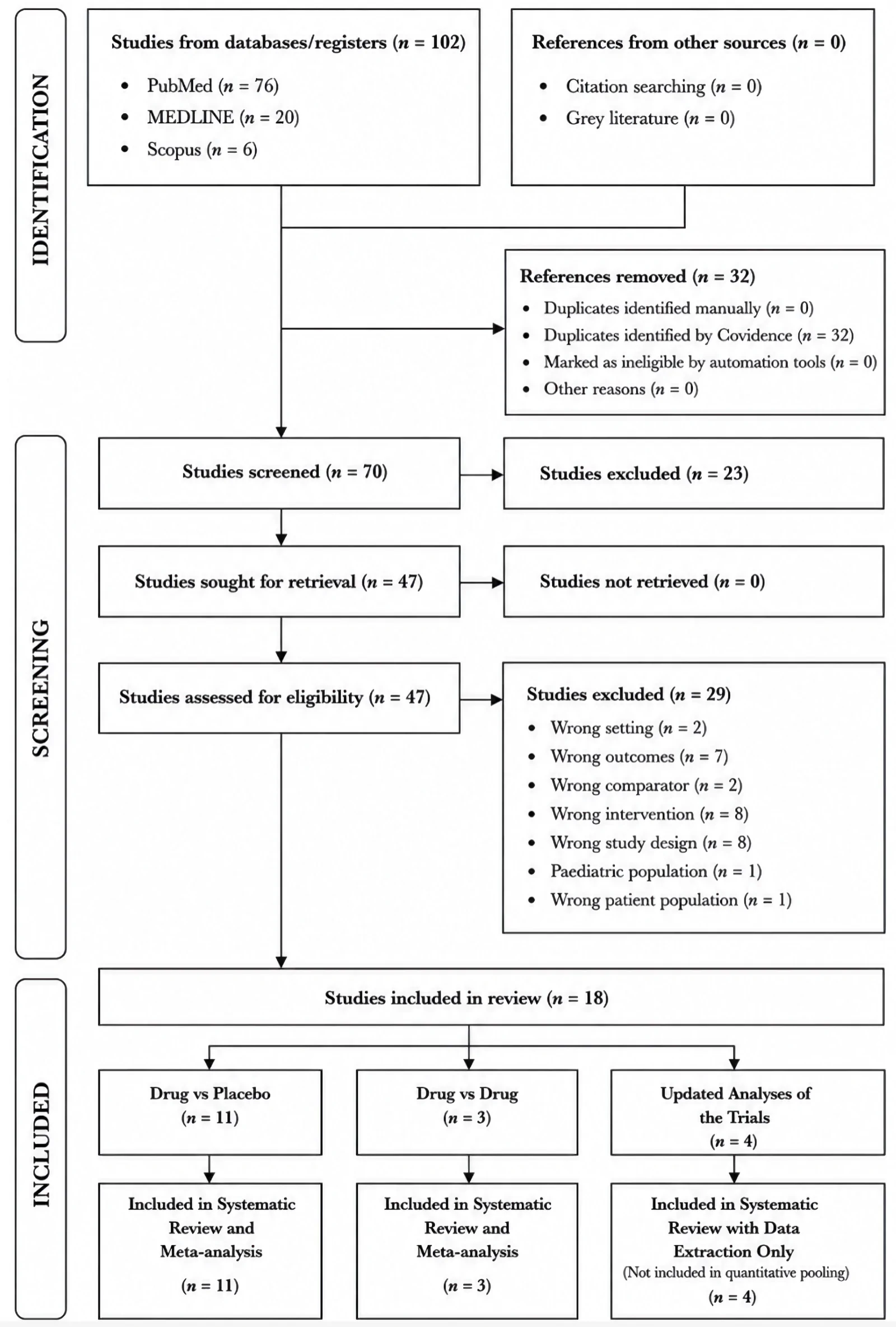
PRISMA flow chart demonstrating search strategy for inclusion of studies reporting randomized controlled trials for tyrosine kinase inhibitors in thyroid carcinoma. PRISMA: Preferred Reporting Items for Systematic Reviews and Meta-Analyses.

Relevant medical subject headings (MeSH) terms, keywords, synonyms, abbreviations, and alternative expressions were incorporated to maximize the sensitivity of the search. The Boolean operator “OR” was used to combine synonymous terms and related concepts within each PICO component, whereas the Boolean operator “AND” was used to combine the different PICO elements to construct the final database search strategy. A comprehensive breakdown of all included studies is provided in the PRISMA flow diagram (Fig. 1).

### Data extraction

Data extraction was performed independently by two researchers using a predefined data extraction form. The following information was collected from each eligible study: first author, year of publication, trial phase, number of participants, patient demographics, thyroid carcinoma subtype, intervention and comparator details, treatment dose, and study characteristics. In addition, efficacy data including hazard ratios (HRs) and corresponding 95% confidence intervals (CIs) for PFS and OS were extracted whenever available.

To facilitate quantitative synthesis, median PFS values for the experimental and comparator groups were also collected. For studies comparing targeted therapies with placebo, PFS data for both the targeted therapy and placebo arms were extracted. Similarly, for drug dose comparison trials, PFS outcomes for both treatment groups were recorded.

A total of 18 studies [25, 32–48] were included in the systematic review, of which 14 contributed to quantitative meta-analysis. Four studies represented updated analyses of the SELECT and COSMIC-311 clinical trials evaluating lenvatinib [43, 44] and cabozantinib [34, 41], respectively. These updated publications were included in the systematic review to provide long-term efficacy data and additional subgroup findings but were excluded from the quantitative synthesis to avoid duplication of patient populations and repeated contribution of the same randomized trial to the pooled effect estimates. Accordingly, Tables 1 and 2 [25, 32, 33, 35–40, 42, 45–47, 48] summarize the baseline characteristics of the placebo-controlled and active-comparator (dose versus dose) randomized trials included in the meta-analysis, whereas Table 3 [34, 41, 43, 44] presents the principal findings from the updated analyses of the SELECT and COSMIC-311 trials that were synthesized descriptively.

**Table 1:** Baseline Characteristics of Placebo-Controlled Targeted Therapy Trials

Study	Registration ID	Malignancy	Phase	Number of patients	Sex of patients	Intervention vs comparator	OS HR (95% CI)	Median follow -up time^a^	Median PFS in months (drug)	Median PFs in months (placebo)	PFS HR (95% CI)
Wells et al, 2012 [37]	NCT00410761	MTC	III	331	Male: 58%/56%	Vandetanib 300 mg vs placebo	0.89 (0.48–1.65)	D (24)/P (24)	30.5	19.3	0.46 (0.31–0.69)
Leboulleux et al, 2012 [48]	NCT00537095	RR-DTC	II	145	Male: 54%/53%	Vandetanib 300 mg vs placebo	0.83 (0.52–1.33)	D (18.9)/P (19.5)	11.1	5.9	0.63 (0.43–0.92)
Elisei et al, 2013 [40]	NCT00704730	MTC	III	330	Male: 68.9%/63.1%	Cabozantinib 140 mg vs placebo	0.98 (0.63–1.52)	D (13.9)/P (13.9)	11.2	4	0.28 (0.19–0.40)
Brose et al, 2014 [39]	NCT00984282	RR-DTC	III	417	Male: 50.2%/45.2%	Sorafenib 400 mg BID vs placebo	0.80 (0.54–1.19)	D (16.2)/P (16.2)	10.8	5.8	0.59 (0.45–0.76)
Schlumberger et al, 2015 [25]	NCT01321554	RR-DTC	III	392	Male: 47.9%/57.3%	Lenvatinib 24 mg vs placebo	0.62 (0.40–1.00)	D (17.1)/P (17.4)	18.3	3.6	0.21 (0.14–0.31)
Schlumberger et al, 2017 [38]	NCT00704730	MTC	III	330	Male: 68.9%/63.1%	Cabozantinib 140 mg vs placebo	0.85 (0.64–1.12)	Not found	Not available	Not available	0.28 (0.19–0.40)
Li et al, 2021 [46]	NCT02586350	MTC	IIB	91	Male: 68%/62%	Anlotinib 12 mg vs placebo	0.92 (0.43–1.97)	D (25.8)/P (24.8)	20.7	11.1	0.53 (0.30–0.95)
Lin et al, 2022 [45]	NCT03048877	RR-DTC	III	92	Male: 41.3%/37.0%	Apatinib 500 mg vs placebo	0.42 (0.18–0.97)	D (18.1)/P (18.1)	20.2	4.5	0.26 (0.14–0.47)
Zheng et al, 2021 [47]	NCT02966093	RR-DTC	III	151	Male: 55.3%/43.8%	Lenvatinib 24 mg vs placebo	0.84 (0.39–1.83)	D (14.8)/P (15.6)	23.9	3.7	0.16 (0.10–0.26)
Brose et al, 2021 [42]	NCT03690388	RR-DTC	III	187	Male: 46%/45%	Cabozantinib 60 mg vs placebo	0.54 (0.27–1.11)	D (6.2)/P (6.2)	Not reached	1.9	0.22 (0.13–0.36)
Leboulleux et al, 2024 [33]	NCT01788982	RR-DTC + MTC	II	101	DTC male: 44.3%; MTC male: 74.2%	Nintedanib vs placebo	DTC: 1.00 (0.41–2.44); MTC: 0.88 (0.24–3.21)	DTC (D (26.3)/P (19.8)); MTC (D (48.4)/P (19.7))	DTC (3.7)/MTC (7)	DTC (2.9)/MTC (3.9)	DTC: 0.65 (0.34–1.25); MTC: 0.49 (0.16–1.53)

^a^D: drug; P: placebo. MTC: medullary thyroid carcinoma; RR-DTC: radioiodine-refractory differentiated thyroid cancer; BID: twice daily; PFS: progression-free survival; OS: overall survival; HR: hazard ratio; CI: confidence interval; DTC: differentiated thyroid cancer.

**Table 2:** Baseline Characteristics of Active-Comparator (Dose vs Dose) Targeted Therapy Trials

Study	Registration ID	Malignancy	Phase	Number of patients	Sex of patients	Intervention vs comparator	OS HR (95% CI)	Median follow -up time^a^	Median PFS in months (drug)	Median PFs in months (drug)	PFS HR (95% CI)
Brose et al, 2022 [35]	NCT02702388	RR-DTC	II	152	Male: 54.7%/48.1%	Lenvatinib 24 mg vs lenvatinib 18 mg	Not reported	D (12.8)/D (11.2)	24 mg (not reached)	18 mg (24.4)	1.44 (0.76–2.74)
Capdevila et al, 2022 [36]	NCT01896479	MTC	IV	247	Male: 73%/60%	Cabozantinib 60 mg vs 140 mg	1.12 (0.77–1.63)	D (30)/D (30)	60 mg (11)	140 mg (13.9)	1.24 (0.90–1.70)
Lin et al, 2021 [32]	NCT02870569/CTR20160220	RR-DTC	II	35	Not clearly extracted	Donafenib 300 mg vs 200 mg	Not reported	Not reported	300 mg (14.98)	200 mg (9.44)	0.678 (0.298–1.547)

^a^D: drug; P: placebo. MTC: medullary thyroid carcinoma; RR-DTC: radioiodine-refractory differentiated thyroid cancer; PFS: progression-free survival; OS: overall survival; HR: hazard ratio; CI: confidence interval.

**Table 3:** Updated Analysis of SELECT (Lenvatinib) and COSMIC-311 (Cabozantinib) Trials With Key Outcomes

Study	Trial number	Experimental vs comparator	Subgroup/analysis type	PFS findings: drug vs placebo	Key outcome reported
Kiyota et al, 2017 [43]	NCT01321554	Lenvatinib 24 mg/day vs placebo	No RAI uptake: 275 patients	NQ vs 3.7 months	Treatment outcomes were comparable regardless of the RR-DTC definition used, suggesting that different diagnostic criteria identify clinically similar patient populations.
			Disease progression despite RAI avidity: 235 patients	16.5 vs 3.7 months	
			Extensive RAI exposure: 73 patients	18.7 vs 3.6 months	
Gianoukakis et al, 2018 [44]	NCT01321554	Lenvatinib 24 mg/day vs placebo	Overall updated SELECT analysis	19.4 vs 3.7 months; HR 0.24; 99% CI, 0.17–0.35	The updated SELECT analysis confirmed sustained PFS benefit with lenvatinib, with responders showing significantly longer PFS than non-responders.
			Responders vs non-responders	Responders: 33.1 months vs 7.9 months in non-responders	
Brose et al, 2022 [34]	NCT03690388	Cabozantinib 60 mg/day vs placebo	Overall updated COSMIC: 311 analyses	11.0 vs 1.9 months; HR 0.22; 96% CI, 0.15–0.32	Long-term follow-up supported the sustained efficacy of cabozantinib in previously treated RAIR-DTC, while maintaining a consistent safety profile.
Capdevila et al, 2024 [41]	NCT03690388	Cabozantinib 60 mg/day vs placebo	Prior sorafenib only	16.6 vs 3.2 months; HR 0.13; 95% CI, 0.06–0.26	Cabozantinib consistently improved PFS across all treatment history and histological subgroups, indicating that its therapeutic benefit is maintained regardless of prior TKI exposure or differentiated thyroid carcinoma subtype.
			Prior lenvatinib only	5.8 vs 1.9 months; HR 0.28; 95% CI, 0.16–0.48	
			Prior sorafenib + lenvatinib	7.6 vs 1.9 months; HR 0.27; 95% CI, 0.13–0.54	
			Papillary DTC	9.2 vs 1.9 months; HR 0.27; 95% CI, 0.17–0.43	
			Follicular DTC	11.2 vs 2.5 months; HR 0.18; 95% CI, 0.10–0.31	
			Oncocytic DTC	11.2 vs 2.5 months; HR 0.06; 95% CI, 0.02–0.21	
			Poorly differentiated DTC	7.4 vs 1.8 months; HR 0.18; 95% CI, 0.08–0.43	

NQ: not quantifiable; PFS: progression-free survival; DTC: differentiated thyroid cancer; RR-DTC: radioiodine-refractory differentiated thyroid cancer; RAI: radioactive iodine; TKI: tyrosine kinase inhibitor; HR: hazard ratio; CI: confidence interval.

Any discrepancies between the two researchers during data extraction were resolved through discussion and consensus. Following completion of the extraction process, the extracted data were jointly reviewed to ensure accuracy and consistency prior to analysis.

### Risk of bias assessment

The methodological quality of the included studies was assessed using the Cochrane Risk of Bias 2 (RoB 2) tool for randomized controlled trials. The assessment was performed across five domains, including bias arising from the randomization process, deviations from intended interventions, missing outcome data, measurement of outcomes, and selection of the reported results. Each study was classified as having a low risk of bias, some concerns, or a high risk of bias. The overall risk of bias judgement was determined according to the RoB 2 guidance and incorporated into the interpretation of the study findings. The four updated analyses of the SELECT and COSMIC-311 trials were not assessed separately using the RoB 2 tool because they represented extended follow-up analyses of previously included randomized controlled trials rather than independent clinical trials. Consequently, the risk of bias assessment was performed only once for each original randomized trial to avoid duplication of methodological quality assessments. The methodological quality of the included randomized controlled trials was independently assessed by two reviewers using the Cochrane Risk of Bias 2 (RoB 2) tool. Any discrepancies in the domain-specific or overall risk-of-bias judgments were resolved through discussion, and the final assessment was reached by mutual consensus.

### Publication bias assessment

Publication bias was assessed using both graphical and statistical approaches. Funnel plots were generated to visually evaluate the symmetry of the included studies around the pooled effect estimate, with marked asymmetry considered suggestive of potential publication bias. In addition, Egger’s regression test was performed using SAS software to statistically assess funnel plot asymmetry. A two-sided P value of less than 0.05 was considered indicative of statistically significant publication bias.

### Statistical analysis

Statistical analyses were performed using SAS software version 9.4 (Maintenance Release 8; SAS Institute Inc., Cary, NC, USA). Meta-analyses were conducted using a random-effects model to account for potential clinical and methodological heterogeneity among the included studies. Treatment effects were summarized as pooled HRs with corresponding 95% CIs and presented using forest plots. For studies that did not report 95% CIs, the reported CIs were converted to 95% CIs using standard statistical methods to ensure consistency across studies and facilitate quantitative synthesis. Separate meta-analyses and forest plots were generated for studies comparing targeted therapies with placebo and for studies comparing different targeted therapy regimens.

Heterogeneity among studies was assessed using the I^2^ statistic. Publication bias was evaluated through visual inspection of funnel plots. In addition to the pooled analyses, bar charts were generated to illustrate the efficacy of targeted therapies relative to placebo or control groups based on PFS outcomes. Updated analyses of previously published trials for lenvatinib (SELECT trial) and cabozantinib (COSMIN-311) were summarized descriptively and presented separately from the quantitative meta-analysis to avoid duplication of patient populations.

### Ethics statement

This study was based exclusively on data obtained from previously published studies and publicly accessible databases. As no individual patient data were collected and no direct involvement of human participants occurred, ethical approval and informed patient consent were not required.

## Results

### Characteristics of included studies

A total of 13 randomized controlled trials comprising 2,671 patients met the eligibility criteria and were included in the systematic review and meta-analysis. Of these, 10 placebo-controlled trials involving 2,237 patients evaluated targeted therapies against placebo, while three active-comparator trials involving 434 patients compared different targeted therapy regimens or doses. The included studies enrolled patients with radioiodine-refractory differentiated thyroid carcinoma (RR-DTC), MTC, or mixed thyroid carcinoma populations and investigated a range of targeted therapies including vandetanib, cabozantinib, sorafenib, lenvatinib, anlotinib, apatinib, nintedanib, and donafenib. The baseline characteristics of the TKIs vs placebo, and drug vs drug studies are presented in Tables 1 and 2 [25, 32, 33, 35–40, 42, 45–48] respectively.

For the nintedanib trial [33], efficacy outcomes were reported separately for DTC and MTC cohorts. These subgroup findings were summarized descriptively but were considered to originate from a single study. Additionally, for the EXAM trial, PFS data were extracted from the primary efficacy publication by Elisei et al (2013) [40], whereas OS data were obtained from the subsequent long-term follow-up analysis reported by Schlumberger et al (2017) [38]. To avoid duplication of patient populations, these publications were considered to represent a single randomized trial, with the most appropriate source used for each outcome.

Table 3 [34, 41, 43, 44] summarizes the four studies demonstrating the updated follow-up analyses of the SELECT and COSMIC-311 trials. These analyses provided additional evidence on the long-term efficacy of lenvatinib and cabozantinib through extended follow-up and subgroup evaluations based on RR-DTC definitions, treatment response, prior TKI exposure, and histological subtype. Overall, the updated analyses consistently demonstrated sustained PFS benefits across the evaluated subgroups. As these publications represented extended analyses of previously included randomized trials, they were synthesized descriptively and were not included in the quantitative meta-analysis to avoid duplication of patient populations.

### Risk of bias assessment

**Figure 2:**
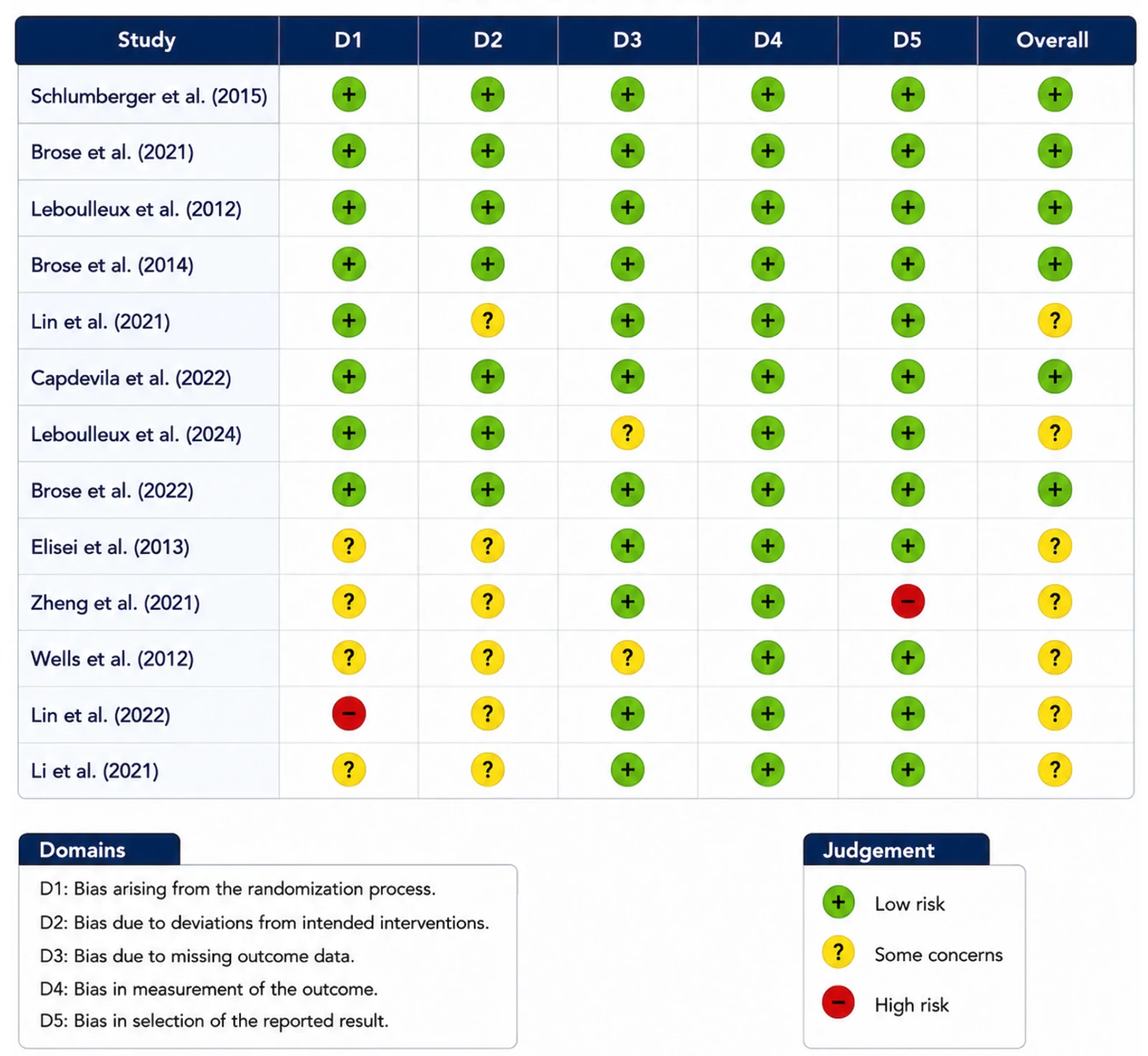
Risk of bias assessment for the included trials.

The methodological quality of the included randomized controlled trials was assessed using the Cochrane Risk of Bias 2 (RoB 2) tool, and the results are presented in Figure 2. Overall, seven of the 13 included studies were judged to have a low risk of bias, while six studies were categorized as having some concerns. No study was at high risk of bias.

The studies judged as having some concerns included Elisei et al (2013) [40], Wells et al (2012) [37], Li et al (2021) [46], Lin et al (2021) [32], Leboulleux et al (2024) [33], and Zheng et al (2021) [47], and Lin et al (2022) [45]. These judgments were primarily attributable to concerns regarding the randomization process, deviations from intended interventions, missing outcome data, or selective reporting. One study (Lin et al, 2022 [45]) demonstrated a high risk of bias in the randomization process, while Zheng et al (2021) [47] showed a high risk of bias in the selection of the reported result. However, these domain-specific concerns did not result in an overall high-risk judgment. Most studies demonstrated a low risk of bias in the domains assessing outcome measurement and deviations from intended interventions, indicating generally robust methodological quality across the included evidence.

### PFS

**Figure 3:**
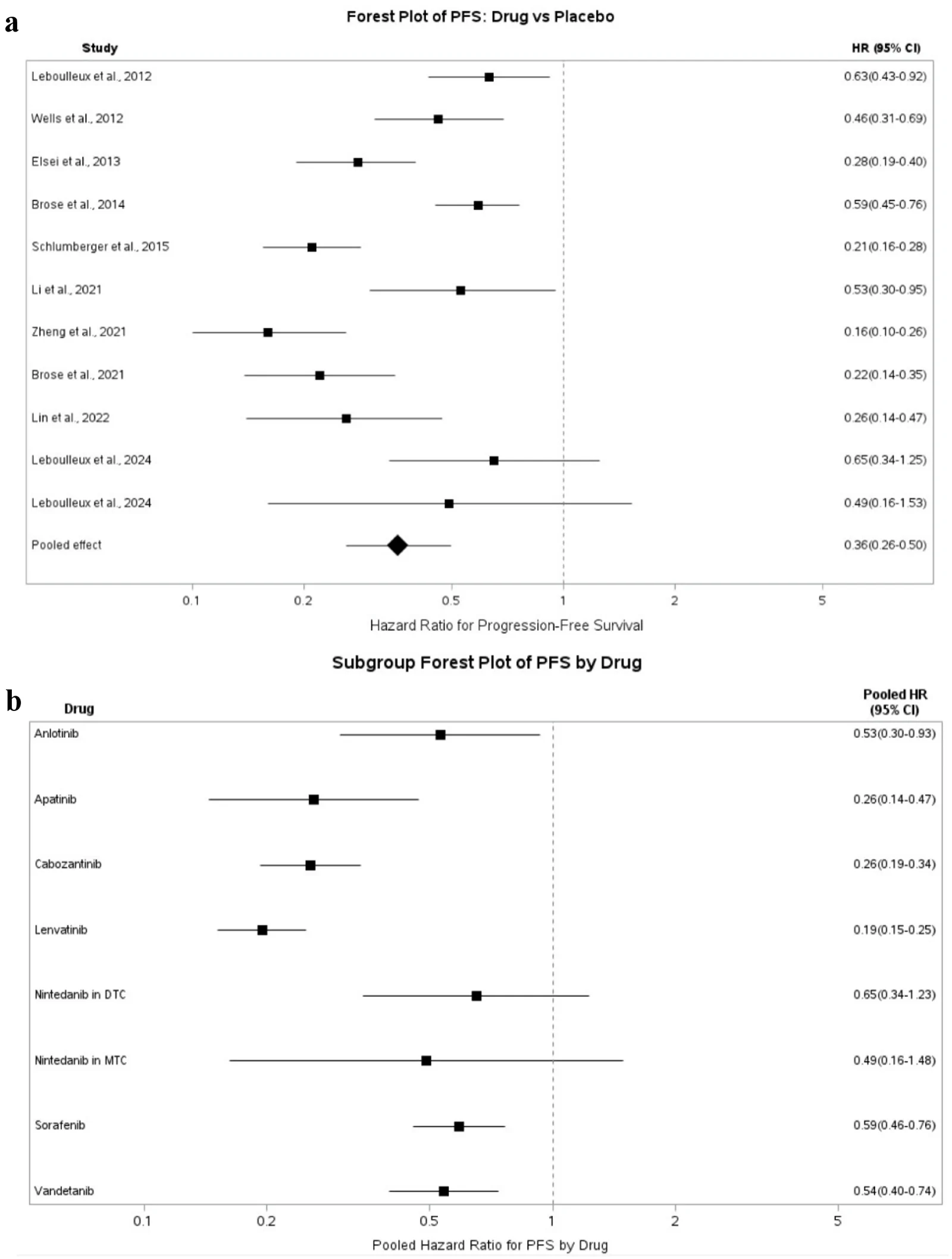
Forest plots of progression free survival (PFS). (a) Drug vs placebo. (b) Drug specific subgroup analysis. HR: hazard ratio; CI: confidence interval; MTC: medullary thyroid carcinoma; DTC: differentiated thyroid cancer.

For the drug versus placebo comparison, 11 PFS effect estimates were included in the random-effects meta-analysis, with the nintedanib trial contributing separate DTC and MTC cohort estimates while originating from a single randomized study. Targeted therapies were associated with a significant improvement in PFS compared with placebo, with a pooled HR of 0.36 (95% CI, 0.26–0.50; z = –6.23, P < 0.001) (Fig. 3a). This indicates an approximately 64% reduction in the hazard of disease progression or death in the targeted therapy arms. However, substantial heterogeneity was observed across studies (Q = 61.56, df = 10, τ^2^ = 0.232, I^2^ = 83.76%). Drug-specific subgroup analysis showed the strongest pooled PFS effects for lenvatinib (HR = 0.19; 95% CI, 0.15–0.25) and cabozantinib (HR = 0.26; 95% CI, 0.19–0.34), while vandetanib, sorafenib, apatinib, and anlotinib also favored targeted therapy (Fig. 3b). Nintedanib showed numerically favorable but statistically nonsignificant PFS effects in both DTC and MTC cohorts. Visual inspection of the funnel plot did not demonstrate strong evidence of publication bias, and Egger’s test did not indicate statistically significant small-study effects (P = 0.103).

For the active drug-dose comparison analysis, three randomized trials were included. The pooled random-effects estimate showed no statistically significant PFS difference between targeted therapy dosing regimens or active comparator arms (pooled HR = 1.18; 95% CI, 0.87–1.60; z = 1.07, P = 0.286). Heterogeneity was low (Q = 2.29, df = 2, τ^2^ = 0.012, I^2^ = 12.55%), suggesting consistency across the included active-comparator trials.

### OS

OS was evaluated using 11 effect estimates from placebo-controlled randomized trials. Consistent with the PFS analysis, the nintedanib study reported separate efficacy outcomes for the DTC and MTC cohorts, which were analyzed descriptively while representing a single randomized trial. In addition, OS data for the EXAM trial were extracted from the long-term follow-up publication by Schlumberger et al (2017) [38], whereas PFS data were obtained from the primary trial publication by Elisei et al (2013) [40] to avoid duplication of patient populations.

**Figure 4:**
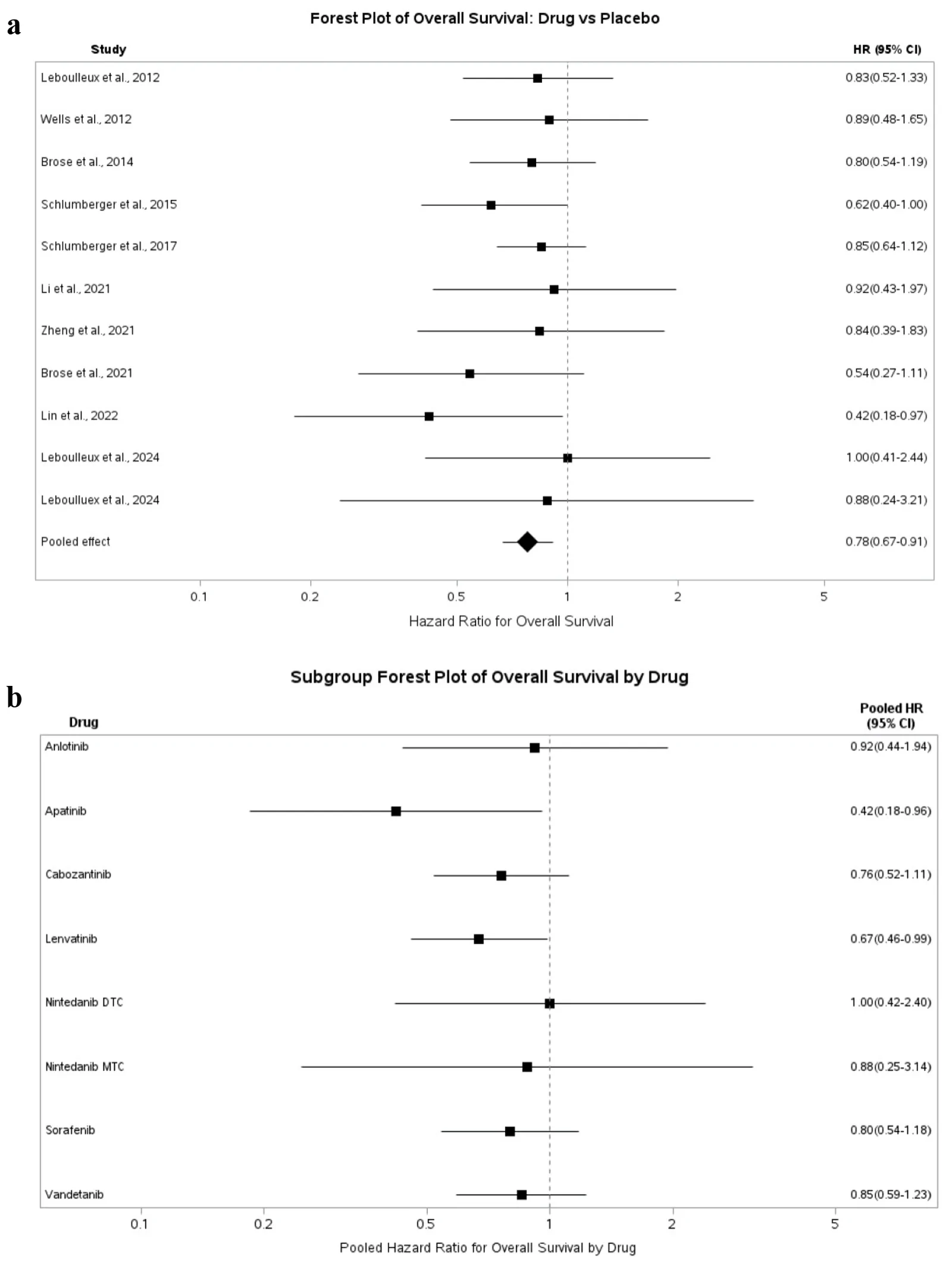
Forest plots of overall survival (OS). (a) Drug vs placebo. (b) Drug specific subgroup analysis. HR: hazard ratio; CI: confidence interval.

The random-effects meta-analysis demonstrated that targeted therapies were associated with a statistically significant improvement in OS compared with placebo, yielding a pooled HR of 0.78 (95% CI, 0.67–0.91; z = -3.14, P = 0.002) (Fig. 4a). This corresponds to an approximately 22% reduction in the hazard of death among patients receiving targeted therapies. In contrast to the PFS analysis, no between-study heterogeneity was observed (Q = 5.46, df = 10, τ^2^ = 0.00, I^2^ = 0.0%), indicating excellent consistency across the included studies. Among individual targeted agents, lenvatinib demonstrated a significant pooled survival benefit (HR = 0.67; 95% CI, 0.46–0.99), whereas cabozantinib showed a favorable but nonsignificant trend (HR = 0.76; 95% CI, 0.52–1.11) (Fig. 4b). Vandetanib, sorafenib, anlotinib, and the nintedanib in the DTC and MTC cohorts did not demonstrate statistically significant improvements in OS, while apatinib showed a significant benefit based on a single study (HR = 0.42; 95% CI, 0.18–0.96). Visual inspection of the funnel plot suggested no substantial asymmetry, and Egger’s regression test did not demonstrate evidence of small-study effects or publication bias (P = 0.323). Overall, these findings indicate that targeted therapies provide a significant survival advantage over placebo, with highly consistent treatment effects across the included randomized trials.

## Discussion

This systematic review synthesized evidence from 18 eligible studies evaluating targeted therapies in thyroid carcinoma. Of these, 14 publications were included in the quantitative meta-analysis, while the risk of bias assessment was conducted across 13 unique randomized controlled trials, as updated follow-up publications arising from the same randomized trial were not assessed separately. Overall, the pooled analyses demonstrated that targeted therapies significantly improved both PFS and OS compared with placebo. Specifically, targeted therapies reduced the risk of disease progression or death by approximately 64% and the risk of death by approximately 22%, supporting their important role in the management of advanced thyroid carcinoma.

This relative benefit is clinically reflected in pivotal randomized placebo-controlled trials: in RR-DTC, median PFS was 18.3 months with lenvatinib versus 3.6 months with placebo in SELECT; in medullary thyroid cancer, median PFS was 11.2 versus 4.0 months with cabozantinib in EXAM, while the predicted median PFS with vandetanib was 30.5 months compared with 19.3 months with placebo in ZETA. These findings demonstrate that the pooled risk reductions translate into clinically meaningful delays in disease progression across advanced thyroid cancer populations.

Although substantial heterogeneity was observed in the PFS analysis, the direction of treatment effect consistently favored targeted therapies across the included studies. This variability is likely attributable to differences in thyroid carcinoma subtypes, molecular targets, treatment doses, previous treatment exposure, and study populations. In contrast, the OS analysis demonstrated excellent consistency with no evidence of statistical heterogeneity, suggesting that the survival benefit associated with targeted therapies was robust across the available randomized evidence. Furthermore, no significant publication bias was identified, and the overall methodological quality of the included trials was high, strengthening the reliability of the pooled findings.

Among the placebo-controlled trials, lenvatinib demonstrated the greatest improvement in PFS, followed by cabozantinib, while vandetanib, sorafenib, apatinib, and anlotinib also showed favorable treatment effects. In the OS analysis, lenvatinib demonstrated a significant pooled survival benefit, whereas apatinib showed a significant survival advantage based on a single randomized trial. Cabozantinib demonstrated a favorable trend toward improved OS, although statistical significance was not reached. Conversely, nintedanib did not demonstrate statistically significant efficacy in either the DTC or MTC cohorts. Furthermore, the active-comparator (dose-versus-dose) analysis did not identify a statistically significant difference in PFS between different targeted therapy regimens or dose strategies, suggesting overall, the PFS benefit was clearly demonstrated in placebo-controlled trials, whereas dose-comparison studies did not show a significant advantage of one targeted therapy regimen over another.

The findings from the dose-comparison analysis are influenced by clinical and methodological heterogeneity across the included studies, including differences in thyroid cancer subtype, study design, sample size, and intervention strategy.

Although pooled PFS did not differ significantly between dosing strategies, the phase III trial by Brose et al [35] demonstrated a significantly higher objective response rate at week 24 with lenvatinib 24 mg compared with 18 mg, suggesting that dose reduction may preserve PFS while reducing early tumor response.

Beyond the quantitative synthesis, four updated analyses of the SELECT and COSMIC clinical trials were reviewed descriptively to capture recently published long-term efficacy data. These analyses provided valuable information regarding the durability of treatment response, long-term PFS and OS outcomes, subgroup-specific efficacy, and extended follow-up beyond the primary trial publications. Although these studies were not incorporated into the meta-analysis to avoid duplication of patient populations, their findings further supported the sustained clinical benefit of lenvatinib and cabozantinib and strengthened the overall body of evidence regarding targeted therapies in advanced thyroid carcinoma. TKI therapy is associated with substantial but generally manageable toxicity; long-term follow-up studies have demonstrated sustained therapeutic benefit with appropriate dose modification and supportive care. Furthermore, consistent efficacy across multiple subgroup analyses suggests that these TKIs provide durable benefit irrespective of prior TKI exposure, histological subtype, or radioiodine-refractory disease definition.

Compared with previous systematic reviews, the present review provides a more comprehensive and contemporary synthesis of the available evidence by incorporating recently published randomized trials evaluating donafenib and nintedanib, together with updated long-term analyses of the SELECT and COSMIC trials. In addition, separate quantitative analyses of placebo-controlled and active-comparator trials allowed a clearer evaluation of treatment efficacy while minimizing clinical heterogeneity. As newer targeted therapies continue to emerge and molecular characterization of thyroid carcinoma evolves, future randomized trials should focus on biomarker-guided patient selection, head-to-head comparisons between targeted agents, combination treatment strategies, and longer-term survival outcomes. Such evidence will be essential for refining personalized treatment algorithms and optimizing the management of patients with advanced thyroid carcinoma.

Despite the encouraging efficacy demonstrated across several targeted agents, additional research remains warranted to further optimize treatment selection. Future randomized trials should focus on direct head-to-head comparisons between targeted therapies, biomarker-guided patient selection, molecular subtype-specific treatment strategies, and longer-term survival outcomes. As novel targeted agents and combination treatment approaches continue to emerge, future evidence syntheses incorporating these therapies will be essential to further refine treatment algorithms and support individualized therapeutic decision-making for patients with advanced thyroid carcinoma.

### Limitations of the review

Several limitations should be considered when interpreting the findings of this review. First, although only randomized controlled trials were included, the number of eligible studies remained relatively limited, particularly for the active-comparator (drug-versus-drug) analyses, thereby reducing the statistical power to detect differences between individual targeted therapies. Second, considerable heterogeneity was observed in the PFS analysis, which likely reflects differences in thyroid carcinoma subtypes, molecular targets, treatment doses, prior therapies, and patient characteristics across the included trials. Although separate analyses were performed for placebo-controlled and active-comparator studies to minimize clinical heterogeneity, these inherent clinical differences may still have influenced the pooled estimates.

Another limitation is that several updated publications from the SELECT and COSMIC trials were not incorporated into the quantitative meta-analysis because they represented extended follow-up analyses of previously included randomized trials. Although this approach was methodologically appropriate to avoid duplication of study populations, some recently reported long-term efficacy data could only be synthesized descriptively rather than quantitatively. Furthermore, direct comparisons between targeted agents were limited because most available randomized trials compared targeted therapies with placebo instead of alternative targeted treatments.

Finally, this review primarily evaluated efficacy outcomes and did not perform a quantitative synthesis of treatment-related adverse events or quality-of-life outcomes. Consequently, the overall benefit-risk profile of individual targeted therapies could not be comprehensively compared. Nevertheless, the inclusion of randomized controlled trials, rigorous study selection following PRISMA guidelines, assessment of methodological quality using the Cochrane RoB 2 tool, separate analyses according to study design, and incorporation of recently published trials and updated long-term analyses strengthen the validity and clinical relevance of the present findings.

## Conclusions

This systematic review and meta-analysis demonstrate that targeted therapies provide significant improvements in PFS and OS in patients with advanced thyroid carcinoma, supporting their important role in contemporary disease management. Among the evaluated agents, lenvatinib demonstrated the most favorable efficacy, while several other targeted therapies also showed meaningful clinical benefit. Nevertheless, the current evidence remains limited by the relatively small number of randomized trials and the lack of direct dosage comparisons between targeted agents. Future well-designed randomized studies with longer follow-up and biomarker-guided treatment strategies are warranted to further optimize personalized management of thyroid carcinoma.

## Data Availability

The data supporting the findings of this study are available from the corresponding author upon reasonable request.

## References

[1] Bray F, Ferlay J, Soerjomataram I, Siegel RL, Torre LA, Jemal A (2018). Global cancer statistics 2018: GLOBOCAN estimates of incidence and mortality worldwide for 36 cancers in 185 countries. CA Cancer J Clin.

[2] Pizzato M, Li M, Vignat J, Laversanne M, Singh D, La Vecchia C, Vaccarella S (2022). The epidemiological landscape of thyroid cancer worldwide: GLOBOCAN estimates for incidence and mortality rates in 2020. Lancet Diabetes Endocrinol.

[3] Sung H, Ferlay J, Siegel RL, Laversanne M, Soerjomataram I, Jemal A, Bray F (2021). Global cancer statistics 2020: GLOBOCAN estimates of incidence and mortality worldwide for 36 cancers in 185 countries. CA Cancer J Clin.

[4] Lubitz CC, Sadow PM, Daniels GH, Wirth LJ (2021). Progress in treating advanced thyroid cancers in the era of targeted therapy. Thyroid.

[5] Baloch ZW, Asa SL, Barletta JA, Ghossein RA, Juhlin CC, Jung CK, LiVolsi VA (2022). Overview of the 2022 WHO Classification of Thyroid Neoplasms. Endocr Pathol.

[6] Capdevila J, Awada A, Fuhrer-Sakel D, Leboulleux S, Pauwels P (2022). Molecular diagnosis and targeted treatment of advanced follicular cell-derived thyroid cancer in the precision medicine era. Cancer Treat Rev.

[7] Romei C, Elisei R (2021). A narrative review of genetic alterations in primary thyroid epithelial cancer. Int J Mol Sci.

[8] Gild ML, Tsang VHM, Clifton-Bligh RJ, Robinson BG (2021). Multikinase inhibitors in thyroid cancer: timing of targeted therapy. Nat Rev Endocrinol.

[9] Xing M (2013). Molecular pathogenesis and mechanisms of thyroid cancer. Nat Rev Cancer.

[10] DeLellis RA, Mangray S (2017). Medullary thyroid carcinoma: a contemporary perspective. AJSP Rev Rep.

[11] Landa I, Cabanillas ME (2024). Genomic alterations in thyroid cancer: biological and clinical insights. Nat Rev Endocrinol.

[12] Lloyd RV, Osamura RY, Kloppel G RJ (2017). WHO classification of tumours of endocrine organs. Fourth Edition - WHO - OMS. WHO classification of tumours of endocrine glands 4th ed.

[13] Filetti S, Durante C, Hartl D, Leboulleux S, Locati LD, Newbold K, Papotti MG (2019). Thyroid cancer: ESMO Clinical Practice Guidelines for diagnosis, treatment and follow-updagger. Ann Oncol.

[14] Hegedus L, Miyauchi A, Tuttle RM (2020). Nonsurgical thermal ablation of thyroid nodules: not if, but why, when, and how?. Thyroid.

[15] Haugen BR, Alexander EK, Bible KC, Doherty GM, Mandel SJ, Nikiforov YE, Pacini F (2016). 2015 American Thyroid Association management guidelines for adult patients with thyroid nodules and differentiated thyroid cancer: the American Thyroid Association Guidelines Task Force on Thyroid Nodules and Differentiated Thyroid Cancer. Thyroid.

[16] Nabhan F, Dedhia PH, Ringel MD (2021). Thyroid cancer, recent advances in diagnosis and therapy. Int J Cancer.

[17] Fugazzola L, Elisei R, Fuhrer D, Jarzab B, Leboulleux S, Newbold K, Smit J (2019). 2019 European Thyroid Association guidelines for the treatment and follow-up of advanced radioiodine-refractory thyroid cancer. Eur Thyroid J.

[18] Nervo A, Gallo M, Sama MT, Felicetti F, Alfano M, Migliore E, Marchisio F (2018). Lenvatinib in advanced radioiodine-refractory thyroid cancer: a snapshot of real-life clinical practice. Anticancer Res.

[19] Molina-Vega M, Garcia-Aleman J, Sebastian-Ochoa A, Mancha-Doblas I, Trigo-Perez JM, Tinahones-Madueno F (2018). Tyrosine kinase inhibitors in iodine-refractory differentiated thyroid cancer: experience in clinical practice. Endocrine.

[20] Kim M, Kim TH, Shin DY, Lim DJ, Kim EY, Kim WB, Chung JH (2018). Tertiary care experience of sorafenib in the treatment of progressive radioiodine-refractory differentiated thyroid carcinoma: a Korean multicenter study. Thyroid.

[21] Berdelou A, Borget I, Godbert Y, Nguyen T, Garcia ME, Chougnet CN, Ferru A (2018). Lenvatinib for the treatment of radioiodine-refractory thyroid cancer in real-life practice. Thyroid.

[22] Balmelli C, Railic N, Siano M, Feuerlein K, Cathomas R, Cristina V, Guthner C (2018). Lenvatinib in advanced radioiodine-refractory thyroid cancer - a retrospective analysis of the Swiss Lenvatinib named patient program. J Cancer.

[23] Brose MS, Nutting C, Jarzab B (2013). Sorafenib in locally advanced or metastatic patients with radioactive iodine-refractory differentiated thyroid cancer: The phase III DECISION trial. Journal of Clinical Oncology.

[24] Brose MS, Nutting CM, Sherman SI, Shong YK, Smit JW, Reike G, Chung J (2011). Rationale and design of decision: a double-blind, randomized, placebo-controlled phase III trial evaluating the efficacy and safety of sorafenib in patients with locally advanced or metastatic radioactive iodine (RAI)-refractory, differentiated thyroid cancer. BMC Cancer.

[25] Schlumberger M, Tahara M, Wirth LJ, Robinson B, Brose MS, Elisei R, Habra MA (2015). Lenvatinib versus placebo in radioiodine-refractory thyroid cancer. N Engl J Med.

[26] Cooper DS, Doherty GM, Haugen BR, Kloos RT, Lee SL, American Thyroid Association Guidelines Taskforce on Thyroid Nodules, Differentiated Thyroid Cancer (2009). Revised American Thyroid Association management guidelines for patients with thyroid nodules and differentiated thyroid cancer. Thyroid.

[27] Valerio L, Pieruzzi L, Giani C, Agate L, Bottici V, Lorusso L, Cappagli V (2017). Targeted therapy in thyroid cancer: state of the art. Clin Oncol (R Coll Radiol).

[28] Orlandi F, Caraci P, Berruti A, Puligheddu B, Pivano G, Dogliotti L, Angeli A (1994). Chemotherapy with dacarbazine and 5-fluorouracil in advanced medullary thyroid cancer. Ann Oncol.

[29] De Besi P, Busnardo B, Toso S, Girelli ME, Nacamulli D, Simioni N, Casara D (1991). Combined chemotherapy with bleomycin, adriamycin, and platinum in advanced thyroid cancer. J Endocrinol Invest.

[30] Viola D, Valerio L, Molinaro E, Agate L, Bottici V, Biagini A, Lorusso L (2016). Treatment of advanced thyroid cancer with targeted therapies: ten years of experience. Endocr Relat Cancer.

[31] Zhao M, Li R, Song Z, Miao C, Lu J (2024). Efficacy and safety of tyrosine kinase inhibitors for advanced metastatic thyroid cancer: A systematic review and network meta-analysis of randomized controlled trials. Medicine (Baltimore).

[32] Lin YS, Yang H, Ding Y, Cheng YZ, Shi F, Tan J, Deng ZY (2021). Donafenib in progressive locally advanced or metastatic radioactive iodine-refractory differentiated thyroid cancer: results of a randomized, multicenter phase II trial. Thyroid.

[33] Leboulleux S, Kapiteijn E, Litiere S, Schoffski P, Godbert Y, Rodien P, Jarzab B (2024). Safety and efficacy of nintedanib as second-line therapy for patients with differentiated or medullary thyroid cancer progressing after first-line therapy. A randomized phase II study of the EORTC Endocrine Task Force (protocol 1209-EnTF). Front Endocrinol (Lausanne).

[34] Brose MS, Robinson BG, Sherman SI, Jarzab B, Lin CC, Vaisman F, Hoff AO (2022). Cabozantinib for previously treated radioiodine-refractory differentiated thyroid cancer: Updated results from the phase 3 COSMIC-311 trial. Cancer.

[35] Brose MS, Panaseykin Y, Konda B, de la Fouchardiere C, Hughes BGM, Gianoukakis AG, Joo Park Y (2022). A randomized study of Lenvatinib 18 mg vs 24 mg in patients with radioiodine-refractory differentiated thyroid cancer. J Clin Endocrinol Metab.

[36] Capdevila J, Klochikhin A, Leboulleux S, Isaev P, Badiu C, Robinson B, Hughes BGM (2022). A randomized, double-blind noninferiority study to evaluate the efficacy of the cabozantinib tablet at 60 mg per day compared with the cabozantinib capsule at 140 mg per day in patients with progressive, metastatic medullary thyroid cancer. Thyroid.

[37] Wells SA, Robinson BG, Gagel RF, Dralle H, Fagin JA, Santoro M, Baudin E (2012). Vandetanib in patients with locally advanced or metastatic medullary thyroid cancer: a randomized, double-blind phase III trial. J Clin Oncol.

[38] Schlumberger M, Elisei R, Muller S, Schoffski P, Brose M, Shah M, Licitra L (2017). Overall survival analysis of EXAM, a phase III trial of cabozantinib in patients with radiographically progressive medullary thyroid carcinoma. Ann Oncol.

[39] Brose MS, Nutting CM, Jarzab B, Elisei R, Siena S, Bastholt L, de la Fouchardiere C (2014). Sorafenib in radioactive iodine-refractory, locally advanced or metastatic differentiated thyroid cancer: a randomised, double-blind, phase 3 trial. Lancet.

[40] Elisei R, Schlumberger MJ, Muller SP, Schoffski P, Brose MS, Shah MH, Licitra L (2013). Cabozantinib in progressive medullary thyroid cancer. J Clin Oncol.

[41] Capdevila J, Krajewska J, Hernando J, Robinson B, Sherman SI, Jarzab B, Lin CC (2024). Increased progression-free survival with cabozantinib versus placebo in patients with radioiodine-refractory differentiated thyroid cancer irrespective of prior vascular endothelial growth factor receptor-targeted therapy and tumor histology: a subgroup analysis of the COSMIC-311 study. Thyroid.

[42] Brose MS, Robinson B, Sherman SI, Krajewska J, Lin CC, Vaisman F, Hoff AO (2021). Cabozantinib for radioiodine-refractory differentiated thyroid cancer (COSMIC-311): a randomised, double-blind, placebo-controlled, phase 3 trial. Lancet Oncol.

[43] Kiyota N, Robinson B, Shah M, Hoff AO, Taylor MH, Li D, Dutcus CE (2017). Defining radioiodine-refractory differentiated thyroid cancer: efficacy and safety of lenvatinib by radioiodine-refractory criteria in the SELECT trial. Thyroid.

[44] Gianoukakis AG, Dutcus CE, Batty N, Guo M, Baig M (2018). Prolonged duration of response in lenvatinib responders with thyroid cancer. Endocr Relat Cancer.

[45] Lin Y, Qin S, Li Z, Yang H, Fu W, Li S, Chen W (2022). Apatinib vs placebo in patients with locally advanced or metastatic, radioactive iodine-refractory differentiated thyroid cancer: the REALITY randomized clinical trial. JAMA Oncol.

[46] Li D, Chi Y, Chen X, Ge M, Zhang Y, Guo Z, Wang J (2021). Anlotinib in locally advanced or metastatic medullary thyroid carcinoma: a randomized, double-blind phase IIB trial. Clin Cancer Res.

[47] Zheng X, Xu Z, Ji Q, Ge M, Shi F, Qin J, Wang F (2021). A Randomized, Phase III study of Lenvatinib in Chinese patients with radioiodine-refractory differentiated thyroid cancer. Clin Cancer Res.

[48] Leboulleux S, Bastholt L, Krause T, de la Fouchardiere C, Tennvall J, Awada A, Gomez JM (2012). Vandetanib in locally advanced or metastatic differentiated thyroid cancer: a randomised, double-blind, phase 2 trial. Lancet Oncol.

